# Monitoring anti-Xa levels and thromboelastography in puerperal venous thromboembolism: a prospective cohort study

**DOI:** 10.3389/fmed.2026.1786254

**Published:** 2026-03-19

**Authors:** Ruijing Ma, Weimin Tao, Wei Luo, Yilu Zhou, Meixiang Yu, Zhendong Xu

**Affiliations:** 1 Department of Anesthesiology and Intensive Care, Shanghai First Maternity and Infant Hospital, Tongji University School of Medicine, Shanghai, China; 2 Department of Pharmacy, Shanghai First Maternity and Infant Hospital, Tongji University School of Medicine, Shanghai, China

**Keywords:** anti-Xa levels, nadroparin, puerperium, thromboelastography, venous thromboembolism

## Abstract

**Background:**

Postpartum venous thromboembolism (VTE) remains a clinical challenge despite thromboprophylaxis. Low molecular weight heparin (LMWH) represents the first-line treatment, but interindividual pharmacodynamic variability complicates dose titration. This study aimed to optimize individualized anticoagulation monitoring based on anti-Xa levels, thromboelastography (TEG) parameters, and biochemical parameters.

**Methods:**

This prospective cohort study included postpartum VTE patients treated with nadroparin at Shanghai First Maternity and Infant Hospital (September 2021–July 2022). Anti-Xa levels, TEG parameters, and coagulation indices were monitored over the initial 10 doses. Patients with peak anti-Xa levels of 0.5–1.0 IU/mL following the 4th dose were classified as the therapeutic group. All participants were followed for 1 year. Efficacy endpoints included anti-Xa levels, coagulation profiles, TEG changes, thrombus resolution, hospitalization duration, and VTE recurrence. Safety endpoints included bleeding, hematoma, prolonged lochia, thrombocytopenia, and skin reactions.

**Results:**

Fifty patients were enrolled. TEG parameters showed a biphasic response: R and K values peaked after four doses, then declined; MA, Angle, and CI exhibited inverse trends. Mean peak anti-Xa levels rose progressively from 0.05 ± 0.08 IU/mL (day 0), reaching 0.59 ± 0.19 IU/mL (day 5). Anti-Xa levels correlated positively with R (*β* = 7.88, *p* < 0.001) and K (*β* = 1.45, *p* = 0.01), and negatively with Angle (*β* = −18.38, *p* < 0.001). No significant differences were observed thrombus or adverse events between groups; but the therapeutic group demonstrated shorter hospitalization (7.54 ± 1.72 vs. 9.04 ± 3.14 days, *p* = 0.044).

**Conclusion:**

Nadroparin shows a nonlinear dose–response in postpartum VTE. Anti-Xa levels and TEG correlations may enhance individualized anticoagulation monitoring strategies.

**Clinical trial registration:**

https://www.chictr.org.cn/bin/project/edit?pid=132010, ChiCTR2100051002.

## Introduction

Venous thromboembolism (VTE), including deep vein thrombosis (DVT) and pulmonary embolism (PE), constitutes the main cause of gestational and postpartum maternal morbidity and mortality in the United States (1). Compared with the general population, pregnant women have a six-fold higher risk of VTE (2), which peaks in the initial 3 weeks postpartum; thus, postpartum PE is common. In recent years, the incidence of VTE-related maternal deaths has increased rapidly in China. Despite thromboprophylaxis, postpartum VTE persists as a major clinical challenge, necessitating optimized anticoagulation monitoring strategies (3).

As recommended by many guidelines, low molecular weight heparins (LMWH) are preferred for women with a higher gestational and postpartum risk of VTE (4) and has been confirmed as effective and safe for gestational VTE prophylaxis or treatment (5). However, interindividual variability in LMWH response, influenced body weight, renal function, and pregnancy-related physiological changes, complicates dosing (6). Anti-Xa levels monitoring has been proposed to guide LMWH therapy (7), but its clinical utility remains debated. Contradictory results and adverse events, such as thrombosis and bleeding complications, have been reported even when the mean peak anti-Xa was within the target range (0.5–1.0 IU/mL) (8). This suggests that anti-Xa levels alone may not fully capture the haemostatic balance, particularly in high-risk obstetric patients (9).

Thus far, many other monitoring parameters have been successfully explored to evaluate the relationship between LMWH and clinical outcomes in prospective randomized studies (10). Thromboelastography (TEG) provides a comprehensive assessment of clot formation, strength, and lysis, offering real-time viscoelastic haemostatic evaluation (11). Recent studies highlighted the usefulness of TEG in assessing the relationship between anti-Xa levels and clinical outcomes in obstetric patients, underscoring the need for more comprehensive monitoring strategies (12). In addition, a TEG-based dosing of enoxaparin for thromboprophylaxis was proposed by a randomized clinical trial (13). While TEG has been widely used in trauma and cardiac surgery, data on the combined use of anti-Xa and TEG in postpartum VTE management are limited (14).

This prospective cohort study aimed to quantify the pharmacodynamic activity of daily subcutaneous nadroparin as VTE treatment in postpartum women with a 1-year follow-up period, with anti-Xa levels, TEG, and biochemical parameters for assessment. By integrating these monitoring modalities, we seek to optimize anticoagulation strategies, reduce thrombotic events, and minimize bleeding risks in this vulnerable population.

## Method

### Study design

This prospective cohort study was conducted at the tertiary-care Shanghai First Maternity and Infant Hospital, with an annual average of 25,000 deliveries. Pregnant women who were diagnosed with postpartum VTE and received nadroparin therapy were enrolled from September 2021 to July 2022. DVT was diagnosed by venous ultrasound, and PE was confirmed by computed tomography pulmonary angiography (CTPA). The exclusion criteria were: previous thromboembolism, heparin allergy, hemoglobin (Hb) < 90 g/L, platelet (PLT) count <50,000 mm^3^, use of oral anticoagulants or antiplatelet agents, and incomplete clinical data that could not be supplemented by follow-up efforts. This prospective cohort study was conducted in accordance with the standards of the Declaration of Helsinki and was approved by the Ethical Committee of Shanghai First Maternity and Infant Hospital (Ethical Committee No. KS21250, date: 2021-8-3) and registered at www.chictr.org.cn (ChiCTR2100051002). All participants provided written informed consent before enrollment. The consent form detailed the study’s purpose, procedures, potential risks and benefits, and the right to withdraw at any time without affecting their standard medical care.

### Clinical management and follow-up

All participants received nadroparin 86 IU/kg, every 12 h, according to the 2018 Chinese Medical Association guidelines for postpartum VTE treatment (15). The peak anti-Xa levels were measured 4 h after nadroparin administration, and the trough anti-Xa levels were quantified before the next nadroparin administration. The peak anti-Xa levels, biochemical parameters, and TEG parameters at the 0th, 2nd, 4th, 6th, 8th, and 10th dose of nadroparin administration were reported. To re-evaluate the thrombotic status, a lower-extremity venous ultrasound or CTPA was performed after 7 days of nadroparin treatment.

After discharge, all patients continued to use nadroparin once daily according to the guidelines, for at least 6 weeks postpartum. Compliance was confirmed through telephone follow-up. Nadroparin does not enter the breast milk, so its use during lactation is safe. All the patients were followed for up to 1 year after delivery, and hematoma, lochia > 6 weeks, thrombocytopenia, and skin allergy and recurrent thrombosis were recorded.

### Data collection

The following clinicodemographic baseline data were obtained from electronic medical records: (1) age, body mass index (BMI), pregnancies; (2) comorbidity, including preeclampsia and gestational diabetes; (3) biochemical parameters, including the anti-Xa levels (reported by the hospital laboratory as absolute values in IU/mL), PLT count, prothrombin time (PT), activated partial prothrombin time (APTT), fibrinogen (FIB), and D-dimer (DD); (4) maternal and perinatal outcomes, including gestational age and mode of delivery; (5) thrombotic state, including thrombosis grading, PE, and DVT; and (6) TEG values, including R, K, MA, CI, ANGEL, and EPL.

### Statistical analysis

This is an exploratory study and the sample size was determined by the available patient volume during the study period. Given the exploratory nature of this study and the lack of prior data for sample size calculation, a convenience sample of 50 patients was enrolled. A post-hoc power analysis was performed upon the completion of the study. Continuous variables are expressed as mean ± standard deviation (SD) and were analyzed using independent *t*-test after normality testing, or with the Mann–Whitney *U* test. Discrete variables are expressed as counts or rates and were analyzed using chi-square or Fisher’s Exact tests. Moreover, linear mixed-effects model analysis was performed to examine the associations between anti-Xa levels and TEG parameters as well as biochemical markers. Two-tailed *p*-values less than 0.05 were considered significant. For each TEG and coagulation parameter, we constructed linear mixed-effects models with anti-Xa levels, group, time, and their interaction term (anti-Xa × group) as fixed effects, and a random intercept for each subject. The model fit was assessed using the Akaike Information Criterion (AIC) and the Intraclass Correlation Coefficient (ICC). Available data at each time point were used without imputation. Linear mixed effects models were fitted using restricted maximum likelihood, which accommodates missing data under the missing at random assumption. No imputation was performed.

## Results

### Participant characteristics

**Figure 1:**
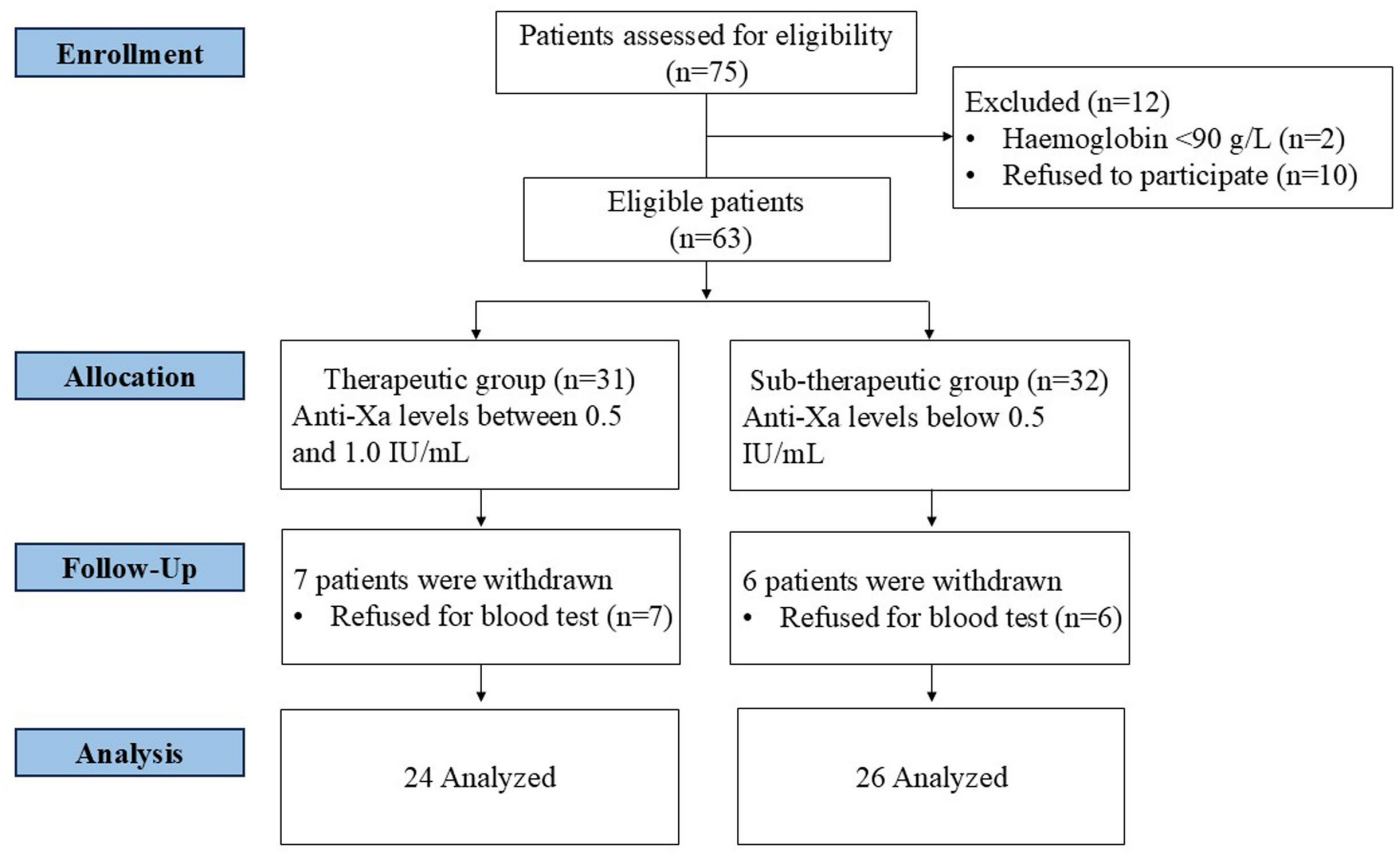
The research flowchart.

The patients’ flowchart is shown in Figure 1. Of 75 women who were eligible in the study. Two women were excluded because of the Hb < 90 g/L, 10 women were excluded because they refused to participate. Moreover, 7 women in the therapeutic group and 6 women in the sub-therapeutic group were excluded because they refused to give a blood test. Fifty patients were enrolled in our study (average age: 33.14 ± 3.63 years, average BMI: 26.81 ± 3.94 kg/m^2^). PE was found in 45 (90.0%) patients and DVT in 13 (26.0%) patients. No patients withdrew from treatment during the initial 10-dose monitoring period (0–5 days of treatment) due to adverse events, poor treatment response or other reasons. All 50 enrolled patients completed the 1-year follow-up with a follow-up completion rate of 100% and a dropout rate of 0%. No patients were lost to follow-up due to migration, loss of contact, death or other factors, and all efficacy and safety endpoints were fully collected for all participants. The Patients were categorized into two groups according to their peak anti-Xa levels at dose 4 of nadroparin: therapeutic group (those with anti-Xa levels between 0.5 and 1.0 IU/mL) and sub-therapeutic group (anti-Xa levels below 0.5 IU/mL). The fourth dose was selected as the grouping time point as it represents a point where steady-state pharmacokinetics are typically achieved, providing a stable and clinically relevant measure of the patient’s anticoagulant response to the fixed-dose regimen. To assess the stability of this classification, we performed a sensitivity analysis using the anti-Xa level after the sixth dose; the results showed that 70% of patients remained in the same group. Sub-therapeutic anti-Xa levels were detected in 26 (52.0%) patients. Table 1 presents the patient characteristics related to therapeutic and sub-therapeutic levels of anti-Xa. The baseline characteristics of the two groups were comparable and showed no statistically significant differences.

**Table 1:** Demographic and pregnancy-related characteristics among VTE patients during the puerperium (*n* = 50).

Variables	Total (*n* = 50)	Sub-therapeutic group (*n* = 26)	Therapeutic group (*n* = 24)	*p*-value
Age, mean ± SD, y	33.14 ± 3.63	32.58 ± 3.50	33.75 ± 3.74	0.26
Weight mean ± SD, kg	70.93 ± 11.40	69.55 ± 14.01	72.46 ± 7.70	0.37
BMI mean ± SD	26.81 ± 3.94	26.39 ± 4.75	27.54 ± 2.60	0.3
Gestational age mean ± SD, d	268.56 ± 18.97	268.46 ± 15.60	268.67 ± 22.40	0.97
Mode of delivery, natural birth	12 (24.00)	4 (15.38)	8 (33.33)	0.14
Parity, *n* (%)				0.55
1	41 (82.00)	20 (76.92)	21 (87.50)	
2	9 (18.00)	6 (23.08)	3 (12.50)	
Gravidity, *n* (%)				0.44
1	27 (54.00)	16 (61.54)	11 (45.83)	
2	12 (24.00)	6 (23.08)	6 (25.00)	
3	11 (22.00)	4 (15.38)	7 (29.17)	
Comorbidities, *n* (%)				
Eclampsia	4 (8.00)	4 (15.38)	0 (0.00)	0.14
GDM	2 (4.00)	2 (7.69)	0 (0.00)	0.49
Thrombotic state, *n* (%)				0.077
PE	5 (10.00)	5 (19.23)	0 (0.00)	
DVT	37 (74.00)	18 (69.23)	19 (79.17)	
PE + DVT	8 (16.00)	3 (11.54)	5 (20.83)	
Grading, *n* (%)				0.68
3–4	34 (68.00)	17 (65.38)	17 (70.83)	
1–2	16 (32.00)	9 (34.62)	7 (29.17)	

Data presented as mean ± SD or number (%). BMI, Body Mass Index; GDM, diabetes mellitus; DVT, deep vein thrombosis; PE, pulmonary embolism.

Parity: Number of deliveries beyond 20 weeks gestation; Gravidity: Number of pregnancies.

### TEG parameters

**Figure 2:**
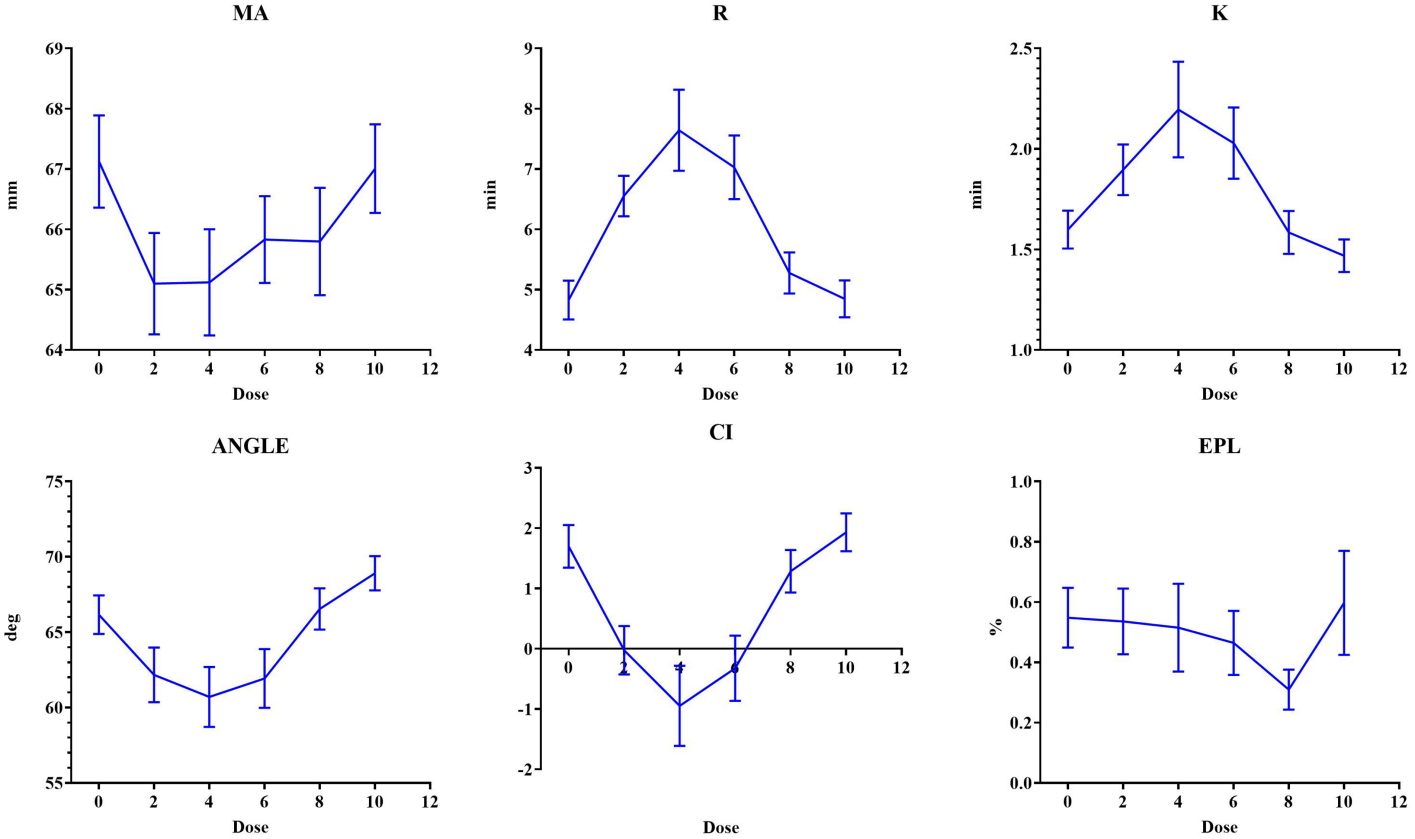
The mean value of TEG parameters across the 10 doses of nadroparin.

**Figure 3:**
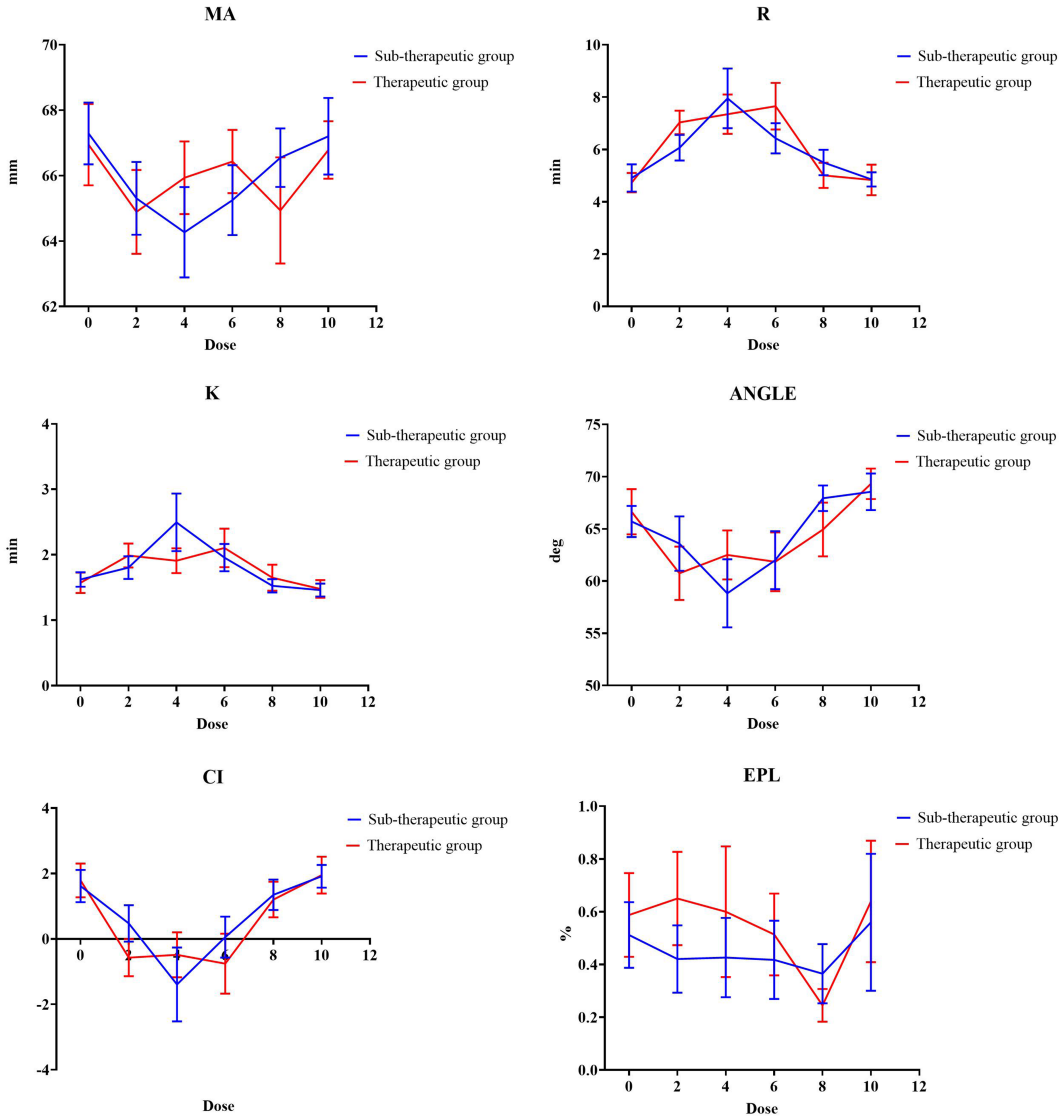
The mean value of TEG parameters in the therapeutic and sub-therapeutic groups across the 10 doses of nadroparin.

The dose–response relationship of the overall TEG parameters (R, EPL, CI, ANGLE, K, and MA) across a dose range of 0–10 is presented in Figure 2. All TEG parameters exhibited a consistent biphasic trend. The parameters MA, Angel, and CI initially showed a decreasing trend but later showed an increasing trend from the 4th to 10th nadroparin doses, with the trough value appearing at the 4th dose. However, the R and K values presented an opposite trend, with the peak value also appearing at the 4th dose. The grouped trends for the therapeutic and sub-therapeutic groups are presented in Figure 3. The TEG parameters of both groups showed biphasic changes. However, the *R* and *K* values in the therapeutic group were consistently higher than those in the sub-therapeutic group, while the Angle value was lower and the sub-therapeutic group maintained higher clot strength throughout treatment.

### Biochemical parameters

**Figure 4:**
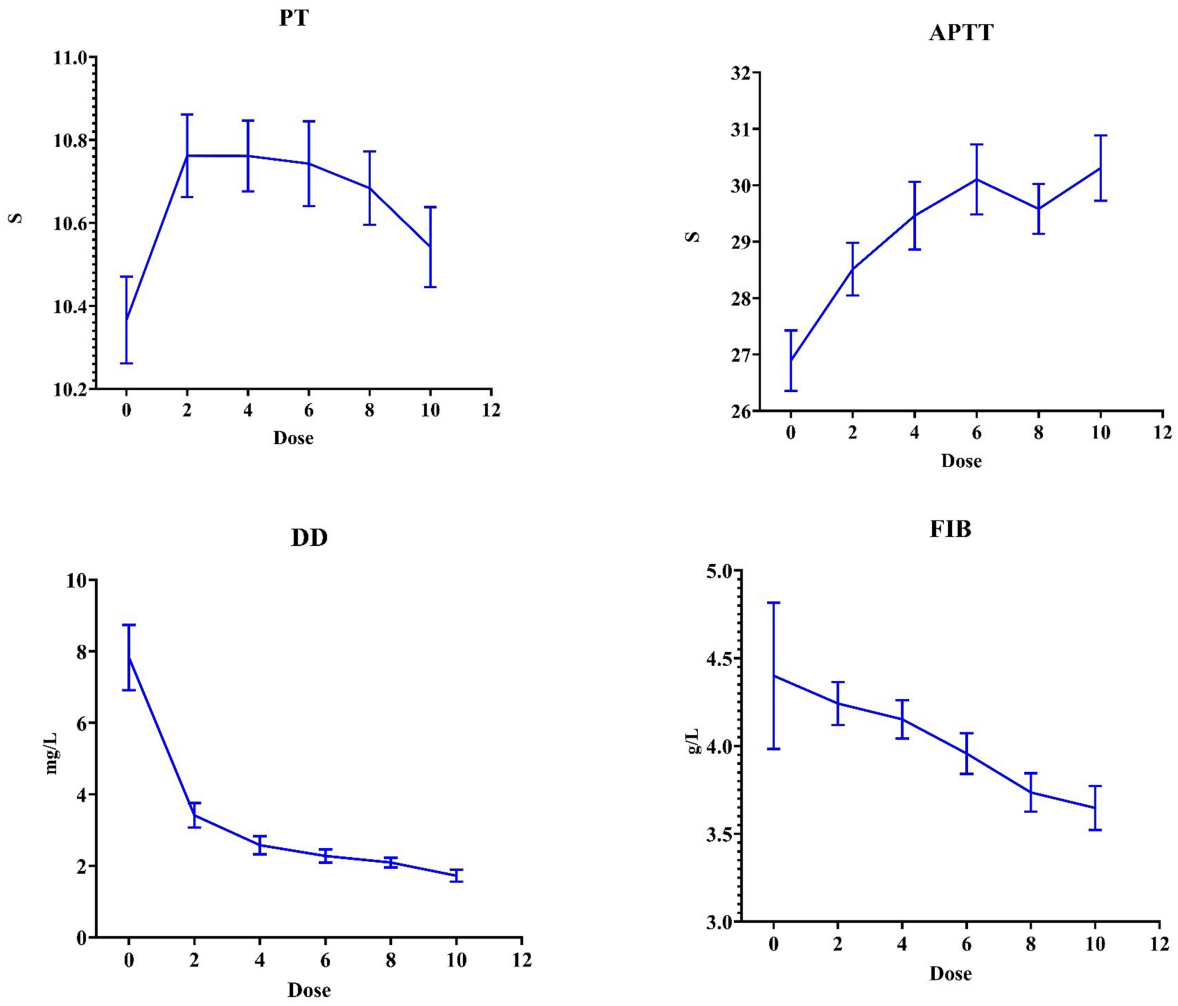
The mean value of coagulation parameters across the 10 doses of nadroparin.

**Figure 5:**
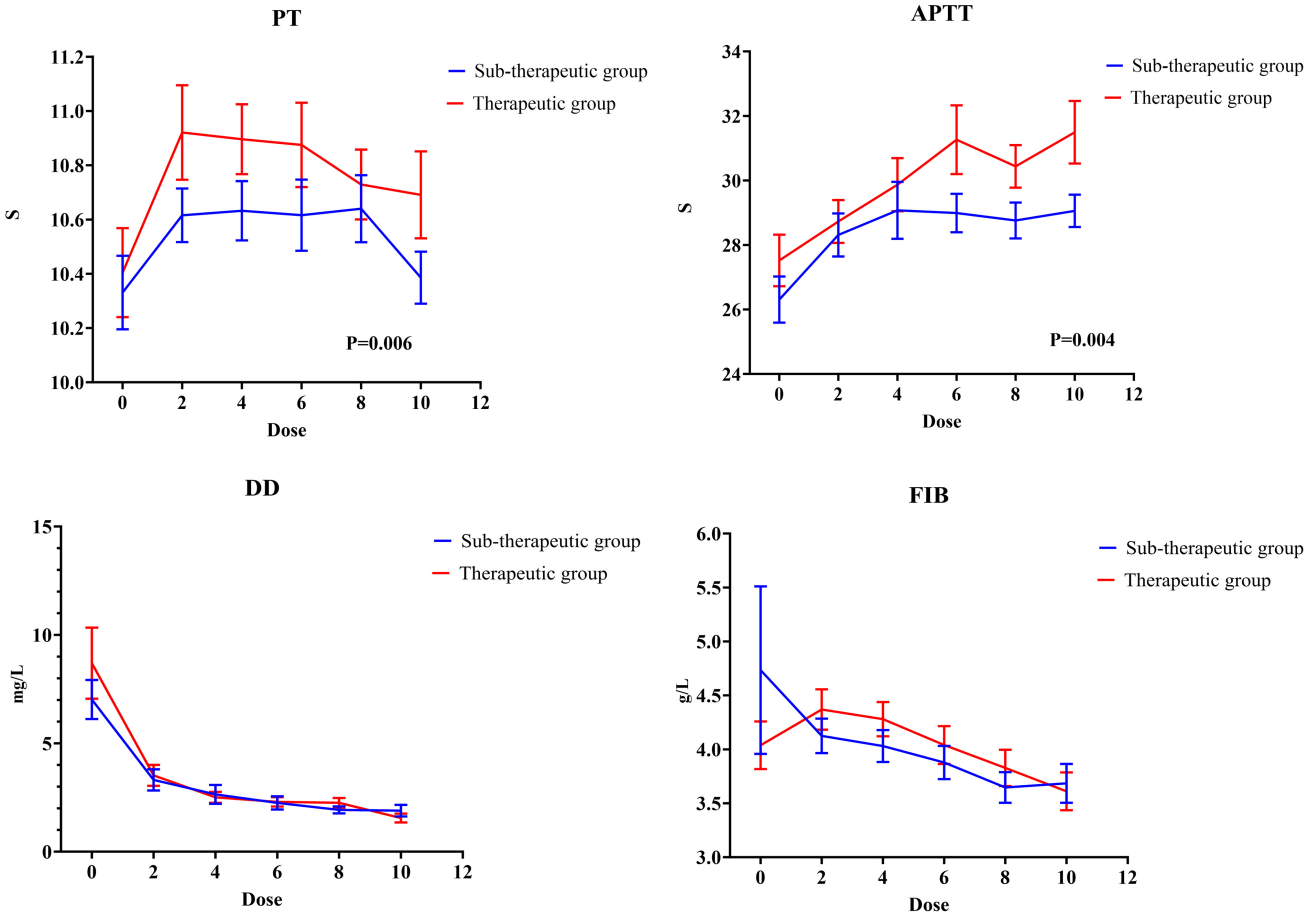
The mean value of coagulation parameters in the therapeutic and sub-therapeutic groups across the 10 doses of nadroparin.

Regarding the coagulation parameters (Figure 4), a dose-dependent decrease in the D-dimer levels (7.82 ± 6.46 vs. 1.66 ± 0.91 mg/L, *p* < 0.001) and FIB (4.40 ± 2.95 vs. 3.66 ± 0.80 g/L, *p* = 0.109) was observed after nadroparin treatment compared with before it. Notably, a plateau phase at 4th dose was observed in the D-dimer levels, suggesting a saturation threshold for receiving nadroparin. On the contrary, an increasing trend of PT and APTT was observed with increasing dose of nadroparin. When stratified by group, the APTT and PT in the therapeutic group were prolonged more significantly, and the D-dimer levels decreased more rapidly. However, the differences between the groups did not reach statistical significance (Figure 5).

### Anti-Xa levels

**Figure 6:**
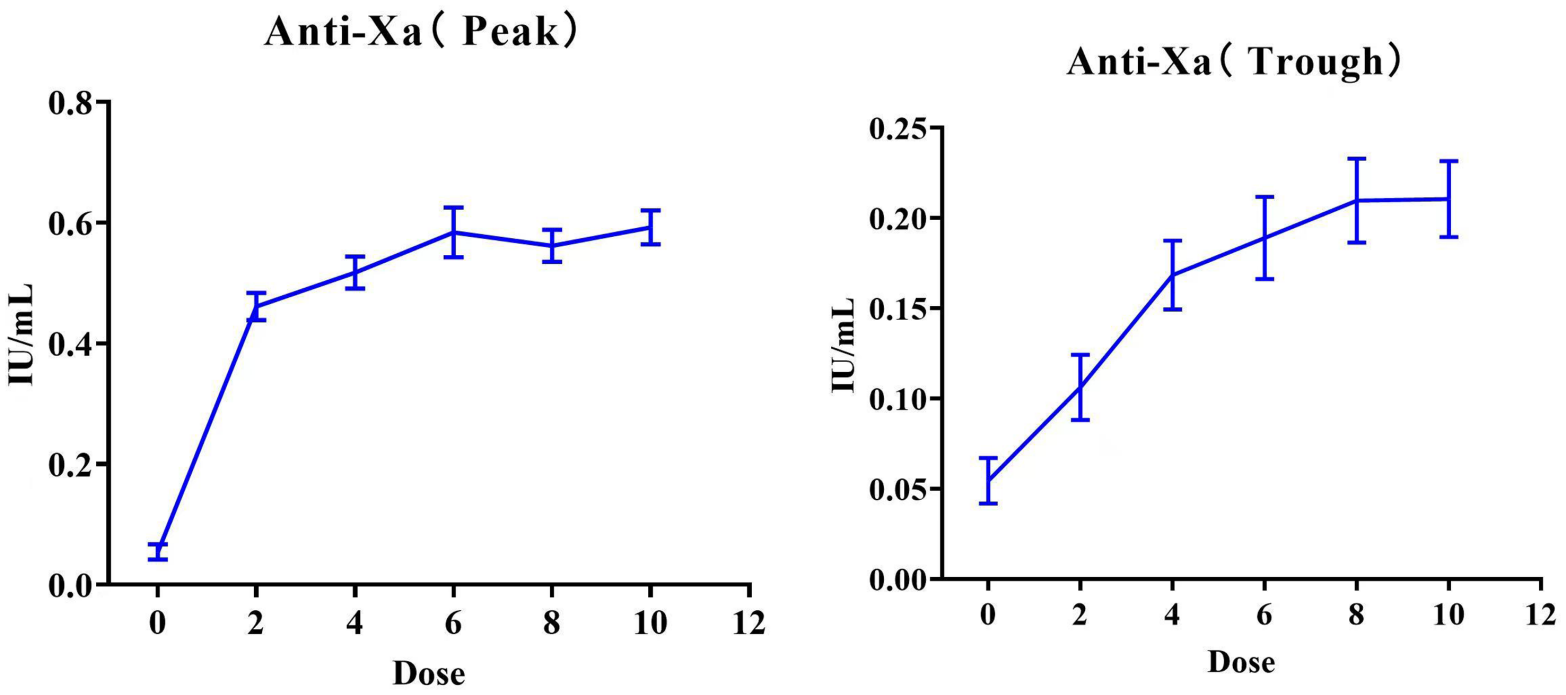
The mean peak and trough anti-Xa levels across the 10 doses of nadroparin.

**Figure 7:**
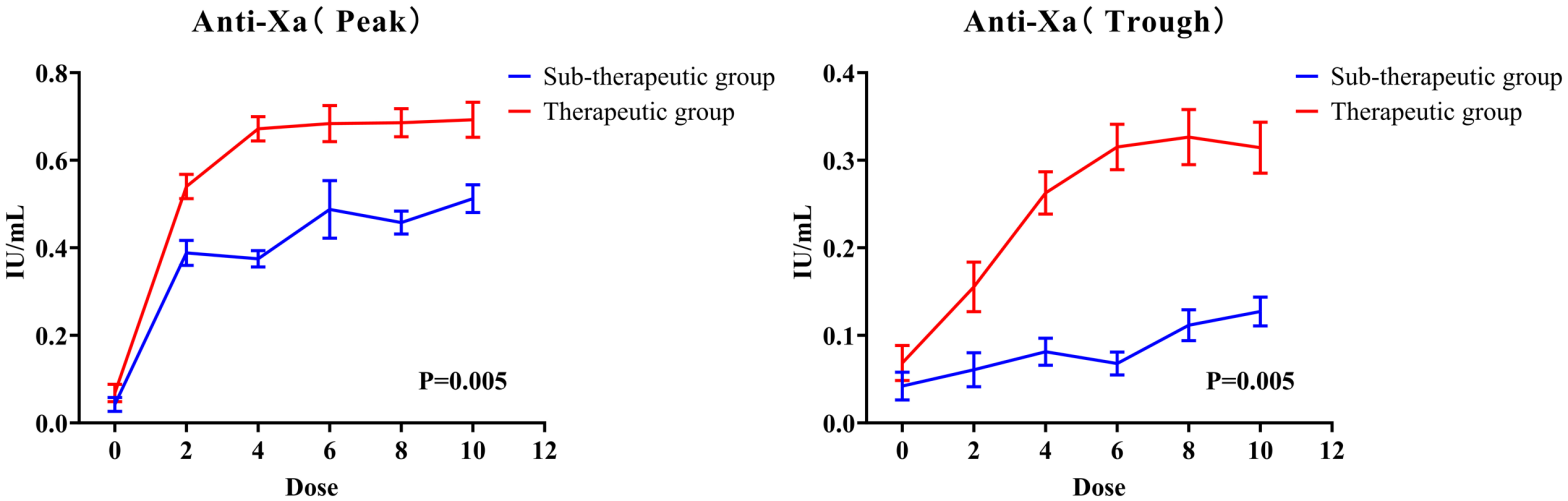
The mean peak and trough anti-Xa levels in the therapeutic and sub-therapeutic groups across the 10 doses of nadroparin.

Figure 6 presents the dose–response relationship of anti-Xa levels at both the peak and trough level after receiving the 10th dose of nadroparin. Both peak and trough levels exhibited a consistent increasing trend, reaching a plateau after dose 4, which corresponds to the steady-state pharmacodynamics of nadroparin. For the peak anti-Xa levels, the mean anti-Xa levels were 0.05 ± 0.08, 0.46 ± 0.16, 0.52 ± 0.19, 0.58 ± 0.29, 0.56 ± 0.18, and 0.59 ± 0.19 IU/mL at the 0th to 10th doses of nadroparin, respectively. Two patients had supra-therapeutic anti-Xa levels at the 4th and 6th doses. Besides, the peak anti-Xa curve displayed a near-linear trend at lower doses (0th–4th doses), with potential saturation effects at higher doses (6th–10th doses). Similar trends were also observed in the trough anti-Xa levels, with an average saturation concentration of approximately 0.2 IU/mL. The rate of achieving a peak anti-Xa levels within target range were 19 (38.00%), 24 (48.00%), 27 (54.00%), 34 (68.00%) and 38 (76.00%) at the 2th to 10th dose of nadroparin. The changes in the anti-Xa parameters between the two groups are shown in Figure 7. The therapeutic group reached a significantly higher peak anti-Xa level, and maintaining levels within the therapeutic range (0.5–1.0 IU/mL) from dose 4 onwards. In contrast, the sub-therapeutic group had a much slower increase, and never achieved the therapeutic target range at any time point.

### Relationship of anti-Xa levels with TEG parameters and biochemical parameters

**Figure 8:**
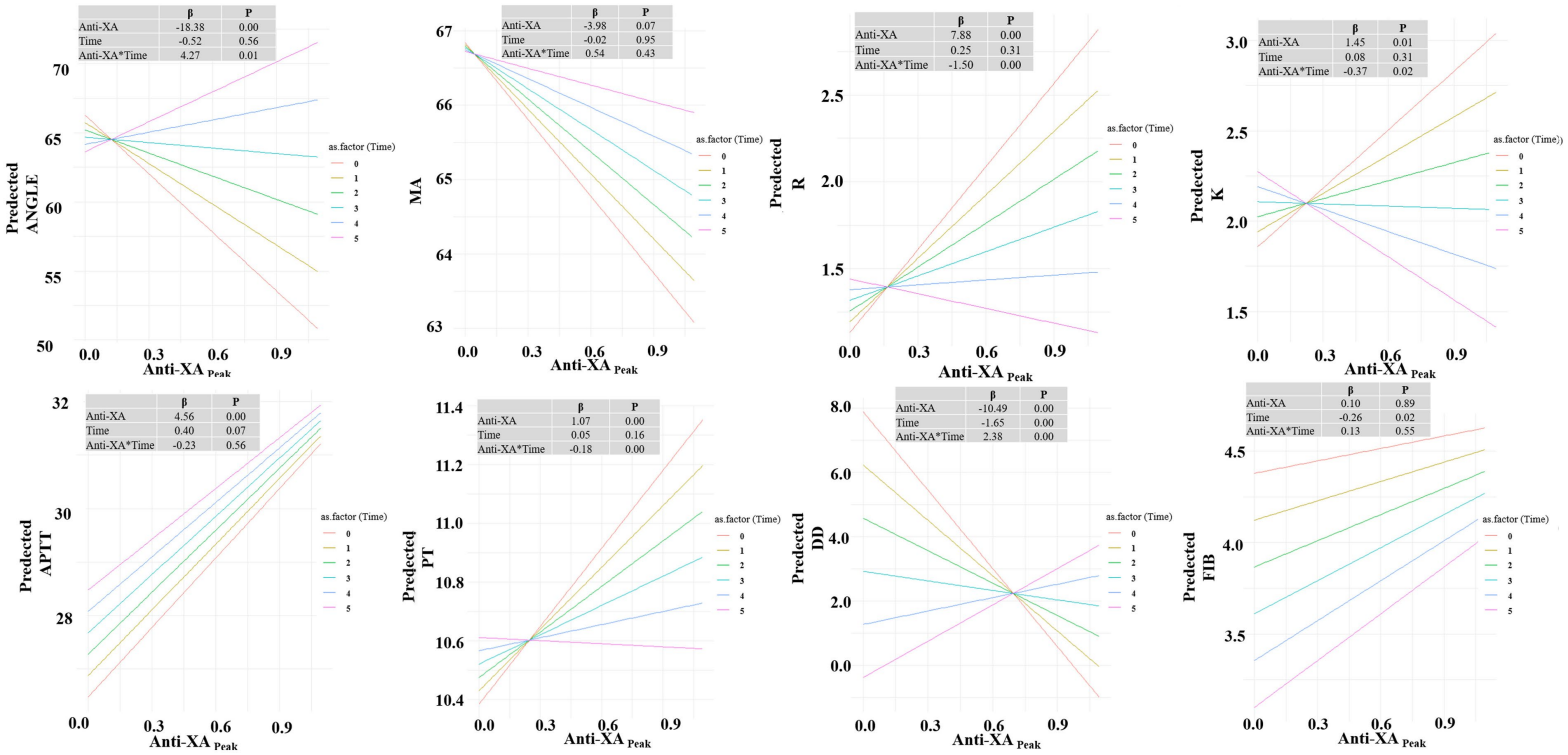
Linear mixed-effects model analysis was performed to examine the associations between anti-Xa levels and TEG parameters and biochemical markers.

**Figure 9:**
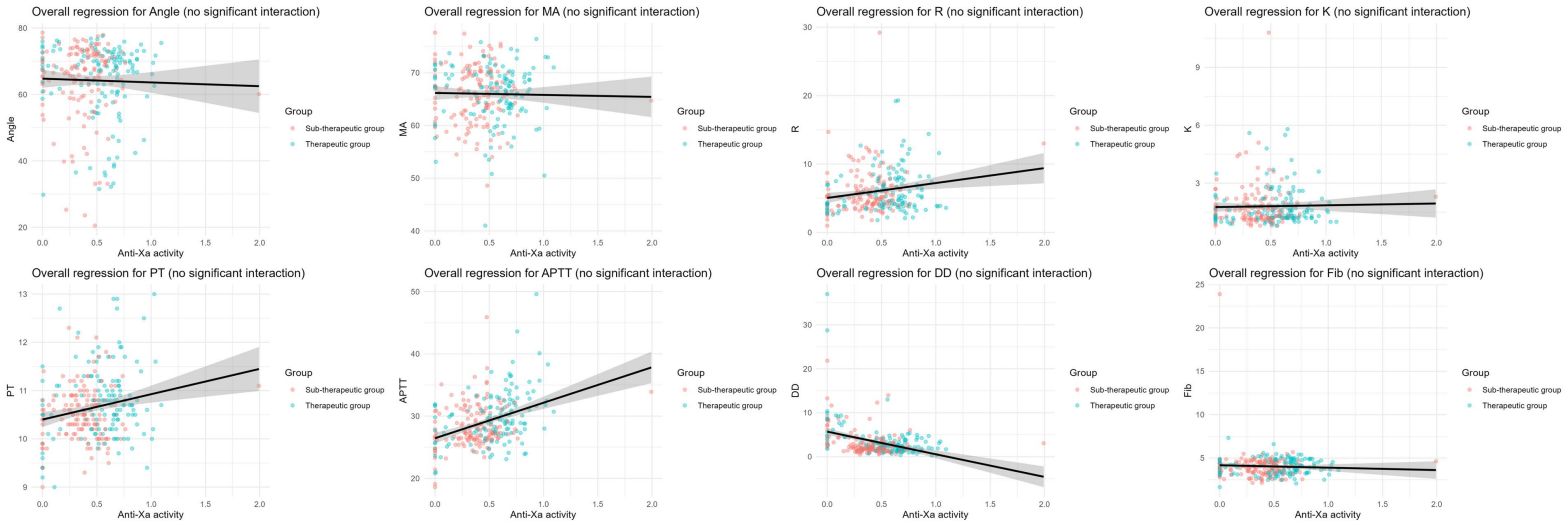
Association between peak anti-Xa levels and coagulation parameters in therapeutic and sub-therapeutic groups.

Linear mixed-effects model analysis was performed to examine the associations of anti-Xa levels with TEG parameters and biochemical parameters (Figure 8). Significant positive associations were observed between anti-Xa levels and TEG parameters R (*β* = 7.88, *p* < 0.001) and K (*β* = 1.45, *p* = 0.01). In contrast, a negative association was found between anti-Xa levels and ANGLE (*β* = −18.38, *p* < 0.001). Regarding biochemical parameters, anti-Xa levels showed significant positive correlations with APTT (*β* = 4.56, *p* < 0.001) and PT (*β* = 1.07, *p* < 0.001). In contrast, a significant inverse relationship was identified between anti-Xa levels and DD levels (*β* = −10.49, *p* < 0.001). No significant associations of anti-Xa levels with MA or FIB were observed. To investigate whether the relationship between anti-Xa levels and coagulation parameters differed between the therapeutic and sub-therapeutic groups, we formally tested for interaction effects by including the term Anti-Xa × Group in our regression models. As illustrated in Figure 9, the regression slopes for anti-Xa against each coagulation outcome were largely parallel between the two groups. This visual observation was confirmed by statistical testing, which revealed no significant interaction effects for any of the parameters analyzed (*p* > 0.05).

The fit of the linear mixed models was assessed using goodness-of-fit indices. The intraclass correlation coefficient (ICC) values for each outcome ranged from 0.11 to 0.72 and the Akaike information criterion (AIC) values ranged from 368 to 2038, suggesting varying degrees of interindividual variability. In the sensitivity analysis, the model fit indices were essentially consistent with those of the full-sample analysis, which further supported the robustness of the results.

### Sensitivity analysis

We conducted a sensitivity analysis, including patients who had completed at least 5 out of 6 time points of anti-Xa levels measurements (*n* = 42). The main findings remained consistent, of which R and K still showed a significant positive correlation and the interaction was not significant.

### Efficacy and safety

After 7-day LMWH treatment, residual thrombus was detected at the original site in 7 (26.92%) and 11 (45.83%) patients in the sub-therapeutic and therapeutic groups, respectively, without any significant intergroup difference (*p* = 0.164). However, the sub-therapeutic group experienced a longer duration of hospitalization (9.04 ± 3.14 vs. 7.54 ± 1.72 days, *p* = 0.044), compared with the therapeutic group.

During the 12-month follow-up, only 3 patients (2 and 1 in the sub-therapeutic and therapeutic groups, respectively) experienced lochia > 6 weeks, and they required no specific treatment. Only two patients experienced postpartum hemorrhage in the sub-therapeutic group. In both groups, the PLT level significantly increased from before to after the 10th nadroparin dose but without any significant intergroup difference (277.48 ± 53.43 vs. 293.70 ± 60.72 10^9^/L, *p* = 0.369). No recurrent VTE, surgical site bleeding, hematoma, thrombocytopenia, or skin allergy were detected during the 12-month follow-up (Table 2). To account for potential confounding, multivariable regression analyses were performed. Linear regression was used for continuous outcomes, and logistic regression was used for binary outcomes (residual thrombus), adjusting for BMI, mode of delivery, PE/DVT classification, and preeclampsia. After full adjustment, anti-Xa levels remained significantly associated with a shorter hospitalization (*β* = −1.83, 95% CI: −3.55 to −0.10, *p* = 0.04), indicating this association was independent of baseline clinical characteristics. The association between anti-Xa levels and residual thrombosis remained non-significant (adjusted OR = 1.06, 95% CI: 0.23 to 4.66, *p* = 0.93), a finding attributed to limited statistical power due to the small sample size. Safety outcomes, including postpartum hemorrhage and hematoma, were too rare to permit meaningful adjusted analysis.

**Table 2:** Efficacy and safety evaluation during the 12-month follow-up.

Variable	Sub-therapeutic group	Therapeutic group	*P*
Efficacy			
Residual thrombus			0.164
No	19 (73.08)	13 (54.17)	
Yes	7 (26.92)	11 (45.83)	
Duration of hospitalization	9.04 ± 3.14	7.54 ± 1.72	0.044
Recurrent VTE			
No	26 (100)	24 (100)	
Yes	0 (0)	0 (0)	
Safety			
Platelet (10^9^/L) at 10th dose	277.48 ± 53.43	293.70 ± 60.72	0.369
Lochia > 6 weeks			1.00
No	24 (92.31)	23 (95.83)	
Yes	2 (7.69)	1 (4.17)	
Postpartum hemorrhage			0.491
No	24 (92.31)	24 (100.00)	
Yes	2 (7.69)	0 (0.00)	
Hematoma			
No	26 (100)	24 (100)	
Yes	0 (0)	0 (0)	
Skin allergy			
No	26 (100)	24 (100)	
Yes	0 (0)	0 (0)	

Values are expressed as mean ± SD or number (%). VTE, venous thromboembolism.

## Discussion

### Principal findings

This study revealed a nonlinear dose–response relationship for nadroparin in treating puerperal VTE, characterized by biphasic trends in TEG parameters and a plateau phase of anti-Xa levels. However, significant positive associations were observed between anti-Xa levels and TEG parameters R and K, and a negative association was found between anti-Xa levels and ANGLE.

### Clinical implications

Low molecular weight heparin (LMWH) has been a focus of research owing to its efficacy and safety in preventing or treating postpartum VTE and the advantage that it neither crosses the placenta nor is secreted in breast milk (16), thereby making it safe for the fetus. Although LMWH has been proven effective and safe in multiple prospective clinical trials, its clinical utility in postpartum remains controversial (17). Based on current evidence, the target steady-state peak and trough anti-Xa measurements are 0.5–1.0 IU/mL or 0.1–0.2 IU/mL, respectively (18). However, previous reports have indicated that the recommended LMWH doses are insufficient for the prophylactic inhibition of Xa in patients with moderate risks for VTE and PE (19). Conversely, a higher-than-recommended LMWH dosage was required to maintain anti-Xa levels in the target range in high-risk patients (20), and another study pointed out that high-dose LMWH was associated with an increased risk of blood loss exceeding 500 mL after vaginal delivery (21). Furthermore, thrombosis occurred even in patients whose anti-Xa levels were within the target range (17). In this study, the mean anti-Xa levels were 0.05 ± 0.08, 0.46 ± 0.16, 0.52 ± 0.19, 0.58 ± 0.29, 0.56 ± 0.18, and 0.59 ± 0.19 IU/mL at the 0th to 10th doses of nadroparin. Overall, 26 (52.00%) and 12 (24.00%) patients at the 4th and 10th doses, respectively, failed to reach the anti-Xa levels within-target (22), which was consistent with other reports. Explanations that may contribute to low anti-Xa target achievement included the following: First, physiological changes in postpartum patients may alter LMWH distribution and pharmacokinetics (23). Second, severities of the postpartum condition are also a significant factor as low early anti-Xa target achievement rate observed in intensive care unit patients (24).

Interestingly, in our study, no significant difference was observed between patients in the sub-therapeutic and therapeutic groups in terms of residual thrombus events and other adverse events. Although this result may be attributed to the limited sample size and short follow-up period, whether the anti-Xa-based LMWH dose adjustment during pregnancy provides any additional benefit was also debated by the International Society on Thrombosis and Haemostasis (25). However, these findings should be interpreted with caution due to the limited sample size and relatively short follow-up period, which may have resulted in insufficient statistical power to detect clinically meaningful differences.

### Research implications

Despite various LMWH treatment strategies, refractory VTE persists. Evidence suggests that anti-Xa levels alone may not fully capture the haemostatic balance, particularly in high-risk patients (26). Many other parameters were explored to predict the efficacy and safety of LMWH, including the coagulation index and TEG parameters (27). One study demonstrated that fondaparinux treatment increased R and decreased MA among TEG parameters (28). Thomas et al. also noticed a significant correlation between TEG parameters and anti-Xa levels (29). The biphasic response of TEG parameters (MA, Angle, and CI) observed in this study, characterized by an initial decline followed by a rebound increase, suggest a dynamic interplay between nadroparin dosage and haemostatic balance. These biphasic trends of TEG parameters were attributed to the effect of LMWH on the fibrinolytic system (30) and platelet function (31). Moreover, in this study, significant positive associations were observed between anti-Xa levels and TEG parameters R and K, and a negative association was found between anti-Xa levels and ANGLE. This result may provide a basis for TEG-guided LMWH dosing, which has been confirmed in the clinical setting by Connelly et al. Anti-Xa levels serve as a specific indicator of the anticoagulant effect of LMWH, accurately reflecting the degree of factor Xa inhibition. However, it cannot assess the overall coagulation balance, which is critical for evaluating the comprehensive coagulation status of patients. In contrast, TEG can comprehensively evaluate the entire coagulation process (32). This unique advantage allows TEG to form a complementary relationship with anti-Xa monitoring.

According to our research results, for patients in the therapeutic group, most of the TEG parameters fall within the expected range. Routine TEG monitoring would result in low cost-effectiveness and no additional clinical value. When combined with high-risk factors (such as obesity, renal dysfunction, or preeclampsia), the anti-Xa levels may not fully reflect the actual coagulation status (33). In such cases, combined monitoring of anti-Xa and TEG can be conducted. For patients in the sub-therapeutic group, TEG monitoring can be combined after the 4th dose to evaluate the overall anticoagulation effect.

### Strengths and limitations

Some limitations of this study that warrant mention are as follows: (1) based on the convenience of LMWH administration, there might be a slight deviation between the actual and predetermined dosage; (2) the sample size was relatively small, and it was conducted at a single center, which may limit the generalizability of the results and the statistical power. Our findings should be interpreted with caution and require validation in larger, multi-center prospective studies; (3) as our data were obtained from a Chinese population that was treated with nadroparin, the results may not be readily generalizable to other ethnicities or settings; (4) fourth, our study has limitations in assessing certain clinical outcomes. The lack of statistically significant differences in residual thrombosis and adverse event rates between groups must be interpreted with caution. Given the sample size is relatively small and the follow-up period is relatively short, the study was likely underpowered to detect differences in these secondary endpoints, particularly for rare events. Therefore, our findings regarding safety and these specific efficacy outcomes should be considered preliminary, and future studies with larger cohorts and longer follow-up are warranted to robustly evaluate these aspects.

## Conclusion

To the best of our knowledge, this is the first prospective cohort study to demonstrate nonlinear dose–response relationship for nadroparin in treating puerperal VTE and significant associations between anti-Xa levels and TEG parameters. These findings underscore the need for individualized monitoring strategies of nadroparin in treating postpartum VTE, combined with monitoring of anti-Xa levels and TEG parameters.

## Data Availability

The raw data supporting the conclusions of this article will be made available by the authors, without undue reservation.
